# Neonatal Gartner Duct Cyst: Two Case Reports and Literature Review

**DOI:** 10.34763/devperiodmed.20172101.3537

**Published:** 2017-05-29

**Authors:** Charu Tiwari, Hemanshi Shah, Jayesh Desale, Mukta Waghmare

**Affiliations:** 1 Dept of Paediatric Surgery, TNMC & BYL Nair Hospital, Mumbai Central, Mumbai, Maharashtra. India. Pin: 400008

**Keywords:** Neonate, Vaginal Mass, Gartner duct cyst

## Abstract

Vaginal cysts are rare, particularly in the newborn. They usually present as one of these three entities in the newborn: paraurethral cysts (Skene duct cysts), Gartner duct cysts (mesonephric ductal remnants) or a covered ectopic ureter. Abdominal ultrasound should always be included in the clinical evaluation in search of renal anomalies. We report two cases of Gartner cysts in neonates.

## Introduction

Vaginal wall cysts are rare in common urological practice, especially in neonates [1]. Gartner duct cysts (GDC) arise as a consequence of obstruction of the Gartner duct (mesonephric remnant). They are located in the anterior or lateral wall of the vagina [1, 2]. They may be associated with renal and ureteral anomalies [1]. The correct treatment is the removal of the entire cyst through a vaginal approach [1]. Aspiration, marsupialisation and deroofing are the various treatment options [1]. Accurate diagnosis can only be made by histology. We report two neonates with Gartner duct cysts aiming to emphasize their embryogenesis, clinical presentations along with differential diagnoses, work-up and management.

## Case studies

**Fig. 1:**
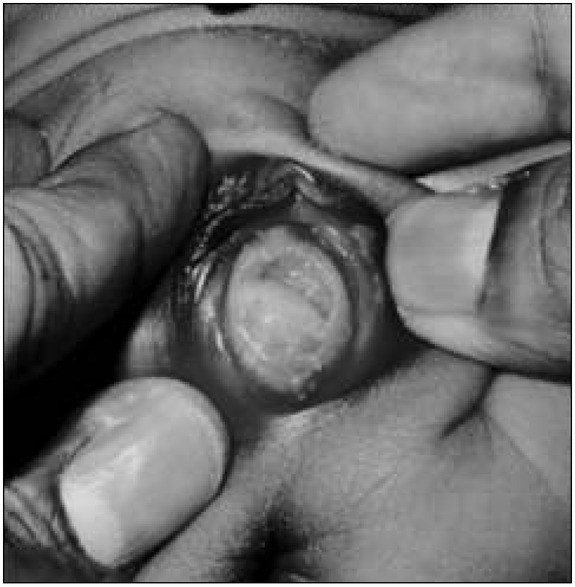
A view of the cystic lesion arising from the right lateral vaginal wall

***Case 1***: A 5-day-old healthy female neonate was admitted with complaints of an asymptomatic vaginal mass, noticed by her mother. General clinical examination was essentially within normal limits. Perineal examination showed normal urethral, vaginal and anal openings. A cystic lesion arising from the right lateral vaginal wall could be seen (Fig. 1).

Abdominal ultrasound was normal. On cystogenitoscopy, the urethra, bladder and both ureteral openings were normal. Vaginoscopy confirmed the cystic lesion in the right lateral vaginal wall. The cyst was deroofed. Histopathology revealed Gartner duct cyst. The patient is asymptomatic on follow-up.

**Fig. 2:**
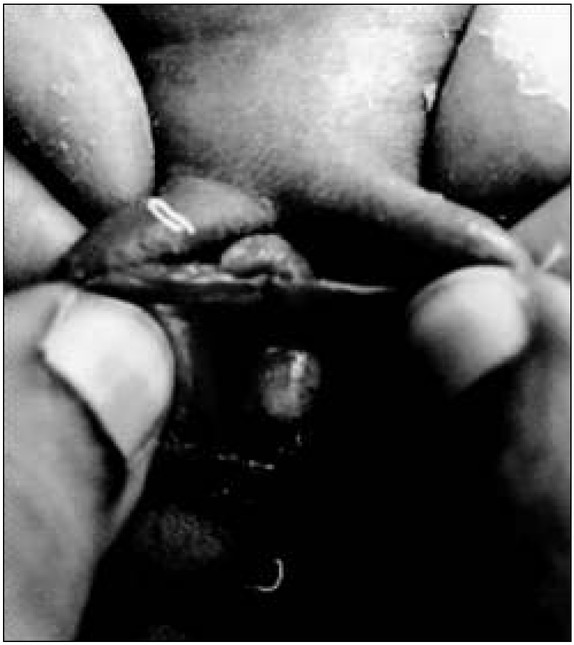
A view of the cystic mass in the left anterolateral wall of the vagina.

***Case 2***: A 6 day-old female neonate was admitted with a swelling over her back. On examination, she had a large lumbosacral rachischisis along with paraplegia and incontinence of the bladder and bowel. On clinical examination of the perineum, the introitus was normal with urethral and vaginal openings. A cystic mass was noted in the left anterolateral wall of the vagina (Fig. 2). The anus was patulous.

Abdominal ultrasound revealed an absent left kidney. On needle aspiration, white milky fluid was aspirated and the cyst collapsed, suggesting Gartner duct cyst.

## Discussion

GDCs represent cystic remnants of the wolfian (mesonephric) duct system and contribute approximately 11% of all vaginal cysts [3]. These develop as a result of incomplete regression of the mesonephric or wolffian duct during fetal development [4]. They are located submucosally along the anteromedial wall of the vagina [2, 4]. Although usually solitary, they may be multiple [4, 5]. The cysts are usually small, less than 2cm in diameter; however, giant cysts have also been reported [5]. The cyst wall is lined with a non-mucin-secreting, squat, cuboidal epithelium [3]. The fluid within the cyst is white, thick, and viscous [3]. Mucoid fluid rules out a mesonephric cyst [3].

If there is a history of antenatal exposure to synthetic hormones, adenosis of the vagina must be considered [4]. The presence of mucosa, which normally stains with Lugol’s solution, helps to exclude the diagnosis of adenosis [4].

GDC is extremely rare in the paediatric age group and more so in neonates [6]. The differential diagnosis of Gartner cyst in a neonate is broad [2]. It includes imperforate hymen (most common), paraurethral Skene’s duct cyst, urethral prolapse, prolapsed ectopic ureterocele, urethral polyp, congenital lipoma, vaginal prolapse, rhabdomyosarcoma of the vagina [1, 7]. Vaginal inclusion cysts and Bartholin gland cysts are usually seen in the third and fourth decades and not in neonates [4].

Accurate diagnosis requires a thorough understanding of the diagnostic possibilities and a systemic evaluation [2]. Physical examination remains the most useful tool for determining the specific pathology [2]. During examination, the patient should be placed in frog-leg position and the labia majora should be gently grasped and pulled caudally and laterally enabling funneling of the introitus and vagina [1]. The size of the clitoris, hymenal configuration, urethral location and the site and character of the mass should be carefully noted [2]. Placing a small feeding tube within the suspected urethral orifice can further help in local examination [2]. Abdominal ultrasound (USG) is a helpful adjunct in diagnosing associated renal disorders [2].

The histology provides the accurate diagnosis on the basis of embryological origin [7]. The vagina is derived from the paramesonephric (Mullerian) duct, the mesonephric (Wolfian) ducts and the urogenital sinus [7]. The vaginal cysts are lined with stratified squamous epithelium as they originate from the Mullerian duct. Gartner duct cysts are lined with cuboidal epithelium {mesonephric/Wolfian origin) [7]. Transitional epithelium in the cyst wall confirms the origin to be in the urinary tract (Skene’s ducts cysts, paraurethral cysts, urethral prolapsed, ureterocoele prolapsed and ectopic ureter) and calls for complete renal work-up [7, 8, 9].

Association of GDC with ipsilateral renal agenesis or dysplasia is rare and is caused by the abnormal development of the ureter [10, 11]. The presence of ureteric ectopia associated with GDC has been reported to be caused by the failure of separation of the ureteric bud from the mesonephric duct, which leads to persistence of Gartner’s duct, frequently with cystic dilation [10, 11]. Presentation with sepsis or non- or poorly functioning renal tissue, is an indication for ureterectomy or nephroureterectomy on the affected side [10].

Management options are aspiration, deroofing, marsupialisation and complete excision [1, 4, 5, 9, 12]. A recent study by Rios et al involving four women has shown that conservative treatment can be a safe option for asymptomatic patients with vaginal GDCs [13]. In a study involving 15 patients by Abd-Rabbo et al, aspiration and injection sclerotherapy with Tetracycline has been reported as one of the management options [14]. This technique has been reported as an ideal, safe and effective simple office procedure for management of symptomatic Gartner cysts [abd14]. However, its application in neonates is unknown. Reassuringly, the long-term prognosis is excellent [12].

## Conclusion

Neonatal Gartner Duct cysts are rare. Association with renal anomalies needs to be ruled out during evaluation. Management is simple and long-term prognosis is excellent.
